# Systems Approach to Human Hair Fibers: Interdependence Between Physical, Mechanical, Biochemical and Geometric Properties of Natural Healthy Hair

**DOI:** 10.3389/fphys.2019.00112

**Published:** 2019-02-21

**Authors:** Elsabe Cloete, Nonhlanhla P. Khumalo, Jennifer C. Van Wyk, Malebogo N. Ngoepe

**Affiliations:** ^1^Hair and Skin Research Lab, Division of Dermatology, Department of Medicine, Groote Schuur Hospital and University of Cape Town, Cape Town, South Africa; ^2^Department of Mechanical Engineering, University of Cape Town, Cape Town, South Africa

**Keywords:** human hair analysis, systems approach, physical properities, mechanical properties, biochemical properties, geometric properties, data reliability

## Abstract

Contextual interpretation of hair fiber data is often blind to the effects of the dynamic complexity between different fiber properties. This intrinsic complexity requires systems thinking to decipher hair fiber accurately. Hair research, studied by various disciplines, follows a reductionist research approach, where elements of interest are studied from a local context with a certain amount of detachment from other elements or contexts. Following a systems approach, the authors are currently developing a cross-disciplinary taxonomy to provide a holistic view of fiber constituents and their interactions within large-scale dynamics. Based on the development process, this paper presents a review that explores the associated features, interrelationships and interactive complexities between physical, mechanical, biochemical and geometric features of natural, healthy hair fibers. Through the review, the importance of an appropriate taxonomy for interpreting hair fiber data across different disciplines is revealed. The review also demonstrates how seemingly unrelated fiber constituents are indeed interdependent and that these interdependencies may affect the behavior of the fiber. Finally, the review highlights how a non-integrative approach may have a negative impact on the reliability of hair data interpretation.

## Introduction

A human hair is a complex biological structure, composed of multi-scale constituents that describe the fiber’s character. At the main structural level, a single hair is divisible into a growing portion and a keratinised portion. The growing portion is generated in the follicle sac embedded in the scalp, whereas the keratinised part protrudes through the scalp’s surface. Each part is a complex structure, having different physical, mechanical, biological, biochemical, and geometric characteristics. Owing to the complexity of the whole hair, main schools of research generally focus on one or two aspects that pertain to a distinct portion. For example, trichology and bioengineering sciences concentrate predominantly on physical and mechanical properties of the keratinised part, whereas biological sciences investigate genetics and biological pathways of the growing portion, and forensic sciences are interested in biochemical and geometric characteristics of the keratinised portion. As a result, datasets obtained from hair studies tend to be disparate, existing as silos of information.

Hair data is exploited across different disciplines. Pathology, dermatology, forensics, anthropology, environmental toxicology, trichology and cosmetology are all engaged in aspects of hair data. Local context engagement is typical of the traditional, reductionist research approach, in which an element of a system is studied with a certain amount of detachment from other elements of the same system. This type of isolation may introduce flawed interpretations and extrapolations of the data. Post-genomic research birthed interdisciplinary systems approaches to biological sciences. A systems approach disentangles the complexity of system constituents and at the same time illuminates large-scale dynamics (Bard, 2013). This implies that following a systems approach for studying human hair fibers explains individual fiber constituents, their interrelationships and their integration into the whole. Until now, there has been no literature focusing on a systems approach to understanding the complexity and dynamics of hair data.

In order to study a complex system effectively, an appropriate taxonomy is required. Without a robust classification system, intra- and intervariability in system constituents are left undefined. Variations in important hair data (e.g., structural composition, mechanical strength, biochemical and lipid composition, absorption and desorption capability) obtained from different genders, ethnicities or age groups, have been highlighted in several studies (Brima et al., 2006; Ettlinger et al., 2014; Lee et al., 2014; Martí et al., 2016). A non-integrated understanding of the dynamics between these datasets complicates interpretation of hair fiber data and raises concern around the reliability of inferences (Wennig, 2000; Popescu and Höcker, 2007).

Historically, race-based classification (Caucasian, African and Asian) has been widely used in hair research. However, a taxonomy based on racial differentiators is subjective and fails to consider diversity that arises from variability within a race, as well as distinctions emanating from genetics, gender, lifestyle, stress-states, aging, nutrition, drugs or disease. Other taxonomies, based on geometric descriptors only, have been proposed but these still fall short of providing an integrated view on hair data for interdisciplinary purposes (de la Mettrie et al., 2007; Loussouarn et al., 2007; Mkentane et al., 2017).

A discipline that is related to hair, namely wool, follows a robust taxonomy to facilitate exploitation of the material. Hair and wool share many structural resemblances. Wool also has an interdisciplinary focus in that it has many different uses. To illustrate, the main taxonomy of the wool industry technically quantifies and qualifies wool fibers as an essential step before and during processing. Main grading parameters relate to geometric descriptions and viable fiber performances within defined boundaries (Sommerville, 2009). Based on correct classification, wool is well understood and therefore successfully utilized. Since concern with hair goes beyond performance, wool taxonomy is not directly transferable to hair.

Important taxonomy parameters that describe a hair fiber are its micro- and macroscopic properties. The first relates to invisible characteristics, such as biochemical composition, structural arrangements, mechanical properties, genetics, drug influence, *et cetera*. Macroscopic properties are sensory observable attributes that result from the underlying microscopic properties, including characteristics such as shininess, perceived healthiness, diameter, color and curliness. Finding interrelationships between all these features requires systems thinking.

The authors of this paper are developing a taxonomy to provide a more holistic view of hair data across different disciplines. Currently, the application of a systems approach to hair data is still in the exploratory phase. As such, various limitations have been placed on the scope. The hair under consideration is in its natural state and has been obtained from healthy female individuals. In addition, only the keratinised portion of the hair is considered. The main emphasis of this first phase was to identify the essential small systems (fiber constituents), find interdependences between these systems and define their integration into large-scale dynamics. Explicit impact factors fall outside the scope of exploratory work, i.e., the influence of race, genetics, gender, lifestyle, stress-states, aging, nutrition, drugs, and disease. These are to be considered only when the essential shared constituents and their dynamics have been well-described. The terms ‘hair fiber’ and ‘fiber’ are used interchangeably. This paper presents a review that explores the associated features, interrelationships and interactive complexities between physical, mechanical, biochemical and geometric properties of natural healthy hair fibers.

The paper offers several important contributions. It illustrates the importance of an appropriate taxonomy for interpreting hair fiber data. It also highlights how seemingly unrelated fiber constituents are indeed interdependent and that these interdependencies may affect the behavior of the fiber. Finally, it elucidates the potential impact of a non-integrative approach on data reliability, and especially on inferences made from such data.

## Systematic Consideration of the Human Hair Fiber

The fiber is a biological multi-system structure, functioning through interacting subsystems, including a physico-mechano profile, biological structure, geometric system, and biochemical system. The inherent complexity of multi-system structures is reducible by decomposition of the (sub)systems into simpler components and behaviors (Chen et al., 2006).

The first step in network decomposition involves identification of key interacting subsystems. Each subsystem is then iteratively decomposed until only singular entities and their interactions remain. The morphology of the fiber is so well-described in literature, that it has become common subject knowledge. Valuable details and summaries have been published in various studies (Wolfram, 2003, Robbins, 2012; Bhushan, 2013; Wortmann, 2014). Focussing on the objectives of this paper, relevant morphological details are summarized to describe the subsystems, entities and their interactions.

**FIGURE 1:**
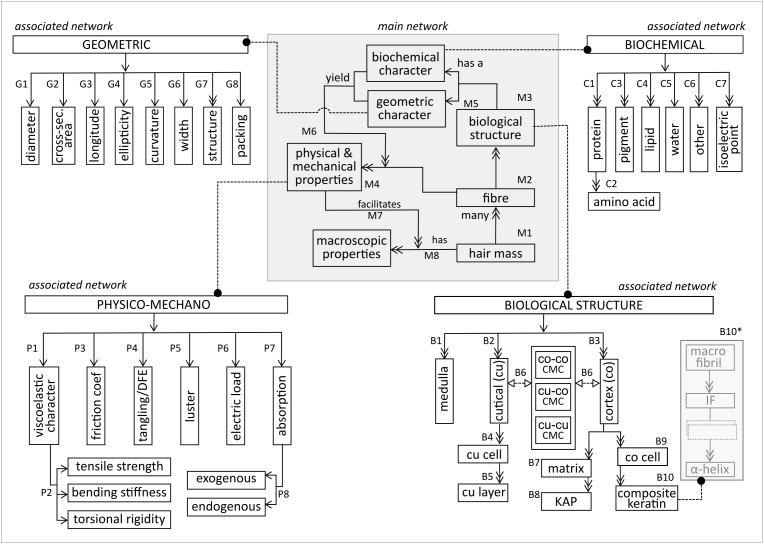
Structural decomposition of the entities in the main and sub networks of the hair fibre.

Figure 1 illustrates the main network, based on the top-level decomposition of the fiber and its associated networks. In the main network, the hair mass (M1) consists of many fibers (M2). Each fiber consists of many biological structures (M3), and has several physical and mechanical properties (M4). Each biological structure has a biochemical and geometric character (M5). These characters combine to lend the fiber its particular physical and mechanical properties (M6). Each fiber has many macroscopic properties (M7), which are generated (M8) by the physical and mechanical properties.

The macroscopic properties (M7) are often used to judge physical attractiveness. Therefore, they appear (to a large extent) to be features that are mostly evaluated subjectively, such as healthy, shiny or dense hair versus dull and thin hair. In relevant studies, certain tactile sensorial properties, which were panel-judged to be ‘unattractive’, have been positively linked to quantifiable properties, e.g., high friction coefficient (Masukawa et al., 2005; Wortmann, 2014). Another relevant example is ‘dull’ hair, which is also deemed as ‘unattractive’. The latter may be the result of haircare product build-up (exogenous absorption), oiliness (excess sebum lipids), degree of curvature, dark pigments or a combination of these. Whilst ‘dull’ may be a subjective description, the mentioned microscopic entities are quantifiable. This means that exploration of the macroscopic properties depends strongly on understanding the microscopic dynamics.

### Biological Structure

The main subsystems of the biological structure network, shown at the bottom right of Figure 1, comprise the medulla, cuticle and cortex (B1–B3). The cuticle, composed of sheet-like cuticle cells [cu cell, (B4)], forms a physico protective barrier for the inner structures. Each cell comprises sublamellar layers [cu layer (B6)], meshed in cell membrane complex (‘CMC’). Besides CMC within the cuticle (cu-cu CMC), CMC also adheres the cuticle to the cortex (cu-co CMC) and meshes together substructures within the cortex (co-co CMC) (B6). Distinction is necessary because the biochemical and structural composition of CMC differs at the various structural locations (Robbins, 2009).

The cortex consists of many cortical cells (B9) embedded in matrix material (B7). The matrix consists of *keratin associated proteins* (KAPs) (B8), which form an interacting globular protein lattice that hosts the cortical cells. A cortical cell is a composite keratin (B10), fundamentally constructed from fibrous α-helix proteins, which aggregate repeatedly into matrix-fused α-helix substructures via disulfide linkages and intermolecular hydrogen bonds (B10^∗^). Within the current scope of model development, focus is placed on the keratin intermediate fiber (IF) and its superstructure, the macrofibril. IF packing within the macrofibril is heavily influenced by the surrounding KAP network which, in turn, influences both fiber rigidity and curvature (Shimomura and Ito, 2005).

### Biochemical Network

As illustrated in Figure 1 (top right), proteins are the dominant biochemical component in the fiber (C1). Each protein is composed of a (poly)peptide backbone formed from amino acids (C2). Other important biochemical components include pigments, lipids, water, and some cellular material and trace elements (C3–6). The isoelectric point (C1.7) is the pH at which the fiber is electrically neutral. Owing to its intricacy, further elucidation of the biochemical associated network warrants an entire study.

### Geometric Network

Geometric descriptors (Figure 1, top left) are multi-scaled, ranging from macro description of the whole fiber to micro- and nano descriptions of the smaller structural units. Mechanically, fibers are often described in terms of their mean diameter (G1), instead of their true diameter, due to the non-uniformity of a fiber across its longitude, as well as polydispersity on the same scalp (Yin et al., 1977). The fiber’s cross-sectional profile (G2) is elliptical rather than circular, with ellipticity ordinarily greater than 1, implying an ellipsoidal rather than a cylindrical longitudinal profile (G3). Ellipticity (G4) is calculated as the ratio between the major and minor elliptical fiber axes. Mathematical integrity is maintained during mechanical calculations that are based on cross-sectional area, irrespective of the fiber’s longitudinal profile. However, knowledge of the true longitudinal profile is essential for mechanical predictions. For example, cylindrical beams are highly resistant to torsion, whereas ellipsoidal beams tend to flatten when subjected to shearing. Hence, a fiber with a high ellipticity is more prone to torsion than one with a low ellipticity. Geometrically, curvature (G5) is the degree of deviation from a straight line, implying zero curvature for straight fibers and higher curvature values for curly fibers. The ‘width’ descriptor (G6) quantifies the thickness of relevant structural units or inter-structural distances. The ‘structure’ descriptor (G7) refers to the shape/form of a structural unit (e.g., primary structure of proteins) and ‘packing’ (G8) is the geometric arrangement of structural units, e.g., hexagonal packing of cortex constituents.

### Physico-Mechano Profile

The physico-mechano network is shown in the top left of Figure 1. The fiber has an inherent viscoelastic character (P1) that describes its deformation capacity under stress, i.e., its tensile strength, bending stiffness and torsional behavior (P2). Owing to the ratio of a fiber’s longitudinal and cross-sectional profiles (geometric network), uniaxial (mechanical) material properties prevail as a result of the strong longitudinal dominance. In addition, most studies have treated the fiber as an elastic, rather than viscoelastic, material. Network decomposition (at the first stage) focussed predominantly on the elastic character.

Friction between two fibers quantifies the effort of sliding over each other. The friction coefficient (P3), which is the ratio of friction between two surfaces in contact and the wedging force, has a proportional relationship with ‘ease of sliding’. Tangling (P4) describes fibers that are interwoven into a lock. Susceptibility to tangling rises when certain factors increase, such as curvature, surface damage, mechanical agitation, wetting and untethered fibers in the hair mass (Wortmann and Schwan-Jonczyk, 2006). Natural detangling tendency is quantifiable by the differential friction effect (DFE), which is the difference between the friction coefficients when rubbing against- and along-scale direction (Wortmann and Schwan-Jonczyk, 2006). This implies that a high DFE value is indicative of untangled hair. Luster (P5) is an inherent material property, referring to the fiber surface’s ability to reflect light specularly. Shininess, rather than luster, is more commonly used as a subjectively judged property of the hair mass. Luster is affected by factors such as illumination source, directions of view and the inherent material gloss-ability, color and curvature (Wortmann et al., 2004). Luster is also affected by acquired fiber attributes such as surface damage, product build-up and electric load (P6) (Wortmann et al., 2004). The fiber’s electric load (P8) refers to the charge on the fiber surface (Velasco et al., 2009), which relates to macroscopic fiber properties, such as combability and fly-away appearance. The fiber absorbs chemicals (P7), with a distinction between exogenous and endogenous absorption (P8). Exogenous absorption is the absorption of water/chemicals from the environment into the fiber, which may be superficial or may penetrate into the deeper layers. Endogenous absorption occurs when chemicals enter the fiber via its growing portion through the bloodstream. Both types of absorption are affected by the biochemical character of the fiber, especially its hydro- or lipophilicity.

## Relationships Between Hair Fiber Entities

**FIGURE 2:**
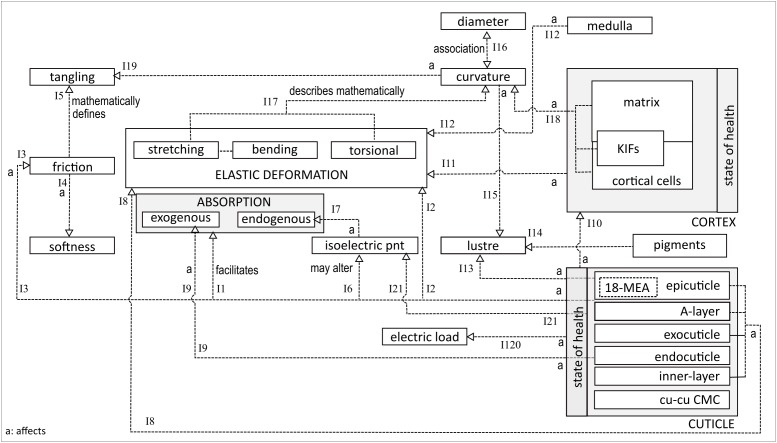
Interaction between different hair fibre properties.

Figure 2 explores the different interrelationships between the entities mentioned above. At the bottom-left is the decomposed structure of a single cuticle cell, comprising sublayers. On the fiber surface, the *epicuticle* layer interacts hydrophobically with the environment, predominantly due to the presence of 18-methyleicosanoic acid (18-MEA). The latter is a fatty acid chain, packed on the surface for optimal density to provisionally facilitate exogenous absorption (I1) (Cheong et al., 2012). 18-MEA is relatively easy to remove or change, either chemically or by high temperature exposure. The result is loss of surface lubrication, leading to an increased tendency of fiber fracture and breaking (I2), and reduced ability of fibres to slide over one another, i.e., increased friction (I3) (Swift, 1999a; Bhushan, 2013). The increased friction coefficient depends strongly on the state of the cuticle. From a macro-perspective, a low friction coefficient facilitates hair softness and smoothness (I4) (Wortmann and Schwan-Jonczyk, 2006). A high friction coefficient is associated with increased resistance to combing, higher potential for damage, inclination to tangling (I5) and a decrease in DFE (Khumalo et al., 2000; Wortmann and Schwan-Jonczyk, 2006).

Changes in 18-MEA concentration may also affect the isoelectric point (I6), hence the fiber’s chemical adsorption and absorption profiles (I7) (Negri and Cornell, 1993). The isoelectric point of a virgin hair fiber is under pH 3.8 (Yin et al., 1977), being kept stable (low) by free lipids on the hair surface (Robbins, 2009). At pH values above the isoelectric point, swelling increases, resulting in an increased rate of diffusion of exogenous chemicals into the hair. Prolonged contact with alkaline products also affects the electric load since it increases the negative charge on the fibers, while acidic protonation increases the positive charge on the surface (I20). Removal of free lipids facilitates the formation of fractures in the cuticle layers. The isoelectric point is, therefore, also associated with the state of the cuticle (I21).

Below the epicuticle are the *A-layer*, exocuticle and inner layers. These have a high cysteine content and a high degree of intermolecular cross-linking (Swift, 1999a,b). Crosslinking and cysteine are the main determinants of the fiber’s mechanical strength and impenetrability. Hence, these layers contribute to the fiber’s hardness and resistance to deformation (I8). The same characteristics also render these layers prone to fracturing under strain. Sandwiched between the exocuticle and inner layer is the endocuticle, a layer with low cysteine and cross-linking (Swift, 1999a). The lack of cross-linking renders it mechanically soft and susceptible to exogenous absorption (I9).

The state of the fiber’s health, in particular the cuticle and cortex, has an impact on other properties. Stretching and bending activities lead to cuticle scale lifting, followed by scale breakage during intra-fiber rubbing (Swift, 1999b). A damaged cuticle increases susceptibility of cortex degradation (I10), resulting in reduced resilience of fibers against mechanical stresses (I11) (Khumalo et al., 2000). Fibers with a medulla have a different inherent density than fibers without a medulla, implying that the presence of a medulla impacts a fiber’s elastic deformation profile (I12) (Merrick, 1998).

The hydrophobic surface of the 18-MEA of the cuticle contributes to the light-reflective properties and hence to the luster of the fiber (I13) (Robbins, 2012). Luster is also affected by hair color (I14) and curvature (I15) (Wortmann et al., 2004). Dark and curved fibers negatively impact luster because dark colors absorb a fraction of the incidental light and significantly curved fibers fail to provide the smooth surface necessary for specular reflection (Wortmann et al., 2004). Curvature has also been shown to be related to diameter based on the composition of cortical cell types (I16) (Orwin et al., 1984). Bearing in mind that a cylindrical or ellipsoidal beam can only change its orientation through bending or twisting if it is to maintain its structural integrity, the geometric phenomenon of curvature can be completely described by Young’s modulus and torsional rigidity (Nissimov and Das Chaudhuri, 2014). This implies that curvature may be expressed geometrically by the fiber’s stretching and torsional deformation profiles (I17). Curvature is strongly influenced by IF- and cortical cell packing and matrix interaction (I18) (Orwin et al., 1984). Curvature also increases the potential for tangling (I19).

Many other relationships have been identified but not elucidated here. Interrelations between biochemical and geometric associated networks have barely been discussed in this paper. Furthermore, exploration of intra-relationships within a single network have also not been shown. However, the essence of Figure 2 reveals the interactive complexity between the different fiber entities which, in turn, illustrates that seemingly unrelated entities affect one another. For example, as indicated earlier, curvature is associated with fiber diameter, tangling, cortical structures, luster and viscoelastic behavior, which is linked to certain cuticle layers, hair lipids and absorption. This implies potential interdependency between curvature and absorption.

Exploration of these entities and their relationships is best facilitated by developing and populating a database. A well-populated set of databases, comprising seemingly unrelated information, is one of the foundational pillars of a systems approach. There are currently no existing databases reported in literature and knowledge on fiber properties is not combined into a single data repository that is shareable amongst various researchers and stakeholders. Instead, access to already existing hair data is only possible by means of literature reviews and individual communications with relevant researchers.

## Conclusion

This review paper demonstrated the first step in identifying relevant entities and their relationships (based on well-described data from literature) to populate a database for hair data. Even though a comprehensive exploration of all fiber constituents and their interdependencies was impractical for this short review, main networks have been described and the intricacy of interdependencies across different networks has been shown. As such, this review drew attention to the potential of an integrative approach to discover previously unknown relationships between fiber features. Awareness of interdependencies between seemingly unrelated fiber constituents implies a holistic and objective view of clinical/experimental data. As a result, inferences made about clinical/experimental hair data are likely to be more robust and reliable.

## Author Contributions

EC, NK, and MN made contributions to the conception and design of the work. NK conceptualized and advocated for this manuscript. EC acquired the data and analyzed the work. EC, NK, JVW, and MN interpreted the work. EC drafted the work. EC, NK, and MN critically revised the work. EC, NK, JVW, and MN provided approval for publication of this content and agreed to be accountable for all aspects of the work.

## Conflict of Interest Statement

The authors declare that the research was conducted in the absence of any commercial or financial relationships that could be construed as a potential conflict of interest.
